# Maternal milk cell components are uptaken by infant liver macrophages via extracellular vesicle mediated transport

**DOI:** 10.1096/fj.202402365R

**Published:** 2025-01-21

**Authors:** Rose Doerfler, Saigopalakrishna Yerneni, Samuel LoPresti, Namit Chaudhary, Alexandra Newby, Jilian R. Melamed, Angela Malaney, Kathryn A. Whitehead

**Affiliations:** ^1^ Department of Chemical Engineering Carnegie Mellon University Pittsburgh Pennsylvania USA; ^2^ Department of Biomedical Engineering Carnegie Mellon University Pittsburgh Pennsylvania USA

**Keywords:** breast milk, digestion, extracellular vesicles, human milk, macrophages

## Abstract

Milk is a multifaceted biofluid that is essential for infant nutrition and development, yet its cellular and bioactive components, particularly maternal milk cells, remain understudied. Early research on milk cells indicated that they cross the infant's intestinal barrier and accumulate within systemic organs. However, due to the absence of modern analytical techniques, these studies were limited in scope and mechanistic analysis. To overcome this knowledge gap, we have investigated the transintestinal transport of milk cells and components in pups over a 21‐day period. Studies employed a mT/mG foster nursing model in which milk cells express a membrane‐bound fluorophore, tdTomato. Using flow cytometry, we tracked the transport of milk cell‐derived components across local and systemic tissues, including the intestines, blood, thymus, mesenteric lymph nodes, and liver. These experiments identified milk‐derived fluorescent signals in intestinal epithelial and immune cells as well as liver macrophages in 7‐day‐old pups. However, the minute numbers of macrophages in mouse milk suggest that maternal cells are not systemically accumulating in the infant; instead, pup macrophages are consuming milk cell membrane components, such as apoptotic bodies or extracellular vesicles (EVs). Ex vivo experiments using primary macrophages support this hypothesis, showing that immune cells preferentially consumed EVs over milk cells. Together, these data suggest a more complex interplay between milk cells and the infant's immune and digestive systems than previously recognized and highlight the need for future research on the role of milk cells in infant health.

## Graphical abstract

Here, a mouse foster nursing model was used to track milk cells in the pup over a period of 21 days. The foster nursing model consisted of wild‐type mice fostered by tdTomato+ dams, enabling maternal cell signal to be visualized in the pups. Flow cytometry data show that infant macrophages consume milk cell components, including extracellular vesicles, rather than maternal cells accumulating in infant organs, suggesting a complex interplay between milk cells and the infant's immune and digestive systems.

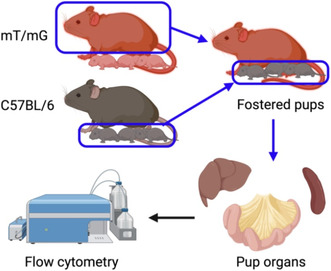

## 1 INTRODUCTION

Milk is a complex food that fulfills the infant's nutritional and developmental needs. Unlike many foods, milk is a living system containing a variety of bioactive components, including antibodies, nucleic acids, maternal cells, and commensal bacteria.^1^, ^2^, ^3^, ^4^ Historically, most research on milk has been nutritional (e.g., fats, sugars), and relatively little attention has been paid to the bioactive and immunological components, especially maternal milk cells.

Among the limited work that has been done, several studies suggest that milk cells have surprising transport properties in the infant. Specifically, some papers have reported that intact maternal cells from milk cross the infant's intestinal barrier and integrate into the tissues of the infant's body. One of the first studies, from 1983, demonstrated that FITC‐labeled mouse milk cells, upon administration to mouse pups, resulted in fluorescent signal in the stomach wall of mouse pups, outside of the digestive tract.^5^ Similarly, in 1984, sheep colostrum cells labeled with FITC produced fluorescent signal in the bloodstream of lambs.^6^ Four years later, Tuboly and colleagues studied the fate of radiolabeled pig colostrum cells and identified radioactive signal in the epithelial layer of the intestine.^7^ In another study, radiolabeled human milk cells were administered to newborn baboons, and radioactive signal was detected in the stomach, intestinal tissues, liver, and spleen of the infants.^8^ This phenomenon is sometimes referred to as breastmilk cell trafficking or breastfeeding‐induced microchimerism.^9^, ^10^, ^11^

As oral drug delivery scientists, we were interested in the transintestinal transport of milk cells because the gastrointestinal tract is typically impermeable to small macromolecules (e.g., peptides). Indeed, our lab and others have spent decades engineering solutions for oral protein delivery.^12^, ^13^, ^14^, ^15^, ^16^ Compared to peptides and proteins, milk cells are 10–100 times larger, and conventional wisdom suggests that they would not permeate the intestine.^17^ Unfortunately, in the 1980s, it was not possible to deduce the mechanism of milk cell transport, given that methods capable of milk cell characterization, such as antibody staining or RNA sequencing, were not broadly available.

To better understand what is known about milk cell transport, we considered more recent literature that used foster nursing mouse models to determine milk cell fate. In foster nursing models, wild‐type mouse pups nurse from a transgenic foster mother expressing a fluorescent protein on all cells.^18^, ^19^ This facilitates the examination of transport phenomena in the infant because milk‐derived cells express the maternal fluorescent reporter protein. Further, the model requires minimal intervention, which allows the mouse pup to have a normal feeding schedule and healthy growth.

Using such foster nursing models, Cabinian and colleagues identified milk‐derived T cells in the Peyer's patches of the intestine, and Aydin et al. reported the presence of milk‐derived stem cells in the brains of mouse pups.^20^, ^21^ Additionally, some studies have asked how milk cell transport impacts the infant's immune system development. For example, one investigation described the role of milk‐derived T cells in developing immunity against specific antigens,^22^ and another detailed the effects of milk cells on delayed‐type hypersensitivity in the infant.^18^ Although these studies made important observations about milk immune cells, the fate of other milk cells and their transport mechanisms remain unclear.

To address this knowledge gap, we employed a foster nursing model and modern analytical techniques to track milk components, including milk cells, in pups up to 21 days of age. We analyzed both local and systemic pup tissues and their individual cell types as a function of pup age. Additionally, we considered the contributions of milk cell‐derived extracellular vesicles in addition to milk cells. Together, our data suggest that a complex transport process is at play and that milk cell signal in pups is attributable not to the transport of maternal milk cells, but instead to infant cells that have taken up milk cell components.

## 2 MATERIALS AND METHODS

### 2.1 Fostering of mice

All mouse experiments were approved by the Institutional Animal Care and Use Committee at Carnegie Mellon University under protocol no. PROTO201600017 and performed in accordance with all institutional, local, and federal regulations. Mice were housed under controlled temperature (25°C) in 12‐h light–dark cycles. Animals were given ad libitum access to a standard diet and water.

mT/mG mice (Jackson Labs) expressing the fluorescent protein tdTomato on all cells and C57BL/6 mice (Charles River) were coordinately mated. Within 24 h after birth, the C57BL/6 pups were transferred to the mT/mG dam. At the time of transfer, the pups were rolled in the bedding of the foster dam's cage in order to mask the scent of the pups and reduce the risk of rejection by the foster mother. The rate of rejection was minimal. We weighed the fostered pups and found that they were healthy and grew at the same rate as nonfostered pups (Figure S1).

At time points up to 21 days of age, the age when mouse pups are typically weaned, the pups were euthanized by CO_2_ asphyxiation followed by decapitation. Blood was collected by cardiac puncture. Organs were collected for analysis by flow cytometry and imaging. Nonfostered C57BL/6 and mT/mG mouse pups were used as negative controls and positive controls, respectively.

### 2.2 Mouse milk collection

To collect milk directly from a lactating mouse, pups were separated from the dam for 2 h prior to milking. The dam was then anesthetized using isoflurane, and 2 IU of oxytocin was injected intraperitoneally to start milk letdown. We had the most success in obtaining mouse milk when two people worked together: one person to manually express milk from the mouse and another person to collect the milk using a 10 μL pipette.

Cells were isolated from the milk as follows: 100 μL of milk was combined with 1 mL of phosphate‐buffered saline (PBS), and the milk was centrifuged for 10 min at 500 × *g*, 4°C. The cell pellet was transferred to a fresh 1.5 mL tube with 1 mL PBS and washed twice by centrifugation for 10 min at 500 × *g*, 4°C, to remove remaining fats and proteins.

### 2.3 Preparation of organs for flow cytometry

Intestines were collected from mice and placed into ice‐cold PBS. Small intestines were flushed with cold PBS using a 25G syringe to wash out intestinal contents. A single suspension of cells was then prepared for flow cytometry according to a modified version of the protocol developed by Couter and Surana.^23^ This protocol was written for adult mice, but some modifications are necessary when working with mouse pups. The authors recommend turning intestinal pieces inside out to expose the epithelial layer. The intestines of mouse pups are much smaller and more fragile than those of adult mice, so it is very difficult to invert the intestinal pieces. Instead, we used a scalpel or razor to cut along the length of the intestine to expose the mucosal surface and then cut the intestine into small pieces.

The epithelial layer was removed by placing the intestinal pieces in a tube of 30 mL RPMI (Roswell Park Memorial Institute) medium containing 500 μL FBS (VWR), 60 μL of 0.5 M EDTA (Sigma), and 93 μL of a 50 mg/mL dithiothreitol stock solution (VWR). After incubating the tube with shaking at 37°C for 15 min, the epithelial cells were collected through a cell strainer, and the remaining intestinal pieces were considered to be the lamina propria. These pieces were washed with RPMI to remove EDTA and then transferred to tubes containing the digest media. For the enzymatic digest of the lamina propria, the intestinal pieces were placed into a solution containing collagenase and dispase. We used lower concentrations of enzymes than those recommended by the protocol because mouse pups have less connective tissue than adults do. For 25 mL of media, we used 20 mg of collagenase II (MP Biomedicals) and 10 mg of dispase II (Sigma). Intestinal pieces were digested in the media for 30 min at 37°C with shaking and then vortexed to break up clumps of cells. Following digestion, the cells were strained through a 70 μm cell strainer (VWR).

The intestinal cells from the epithelial layer and the lamina propria were pelleted by centrifugation for 10 min at 500 × *g*, 4°C. The pelleted cells were re‐suspended in PBS to wash off remaining enzymes and debris and then centrifuged again at 500 × *g*. From there, the single‐cell suspensions were stained for flow cytometry.

Spleens were collected from mice and placed into ice‐cold PBS. A single‐cell suspension was prepared by smashing the spleen through a 70 μm cell strainer using the plunger of a 3 mL syringe. Mechanical dissociation was used here instead of enzymatic because it is simpler, and studies have shown that it is appropriate for our chosen endpoints.^24^ Cells were pelleted for 10 min at 500 × *g*, then re‐suspended in 5 mL of cold red blood cell lysis buffer (VWR). After 5 min of incubation in lysis buffer, cells were re‐suspended in flow buffer and washed twice by centrifugation.

For the thymus and mesenteric lymph nodes, there is no enzymatic digest necessary and no red blood cell lysis step. Again, mechanical dissociation was chosen for the same reasons as above.^25^, ^26^ The most important consideration is the dissection step. Mesenteric lymph nodes, found along the colon, were removed with forceps. Any mesenteric fat was carefully removed, because it can cause additional noise in the sample and reduce cell viability. These organs were smashed through a 70 μm cell strainer using the plunger of a 3 mL syringe, centrifuged for 5 min at 500 × *g*, then re‐suspended in flow buffer and washed once more by centrifugation to prepare a single‐cell suspension.

Livers were digested using a gentleMACS dissociator and the mouse liver digestion kit (Miltenyi Biotech, Auburn, CA), as per the manufacturer's instructions.

Blood was collected from mice by cardiac puncture. The blood was collected into 2 mL EDTA‐coated tubes and placed on ice. Blood was combined with 2 mL red blood cell lysis buffer and incubated at room temperature for 5 min. Then, the cell pellet was quenched with 10 mL PBS and washed twice by centrifugation at 500 × *g* to remove lysed red blood cells and other debris.

In mouse pups that have not yet begun to eat solid food, the milk in the stomach forms a soft curd. To collect milk from the stomachs of pups after euthanasia, a small incision was made in the stomach, and the milk curd was removed with forceps. The milk curd was smashed through a 70 μm cell strainer using the plunger of a 3 mL syringe, centrifuged for 10 min at 500 × *g*, 4°C, re‐suspended in flow buffer, and washed twice by centrifugation.

### 2.4 Fostered mice flow cytometry

Cells were stained with antibodies (Table 1) at a 1:100 dilution on ice for 30 min. All flow cytometry was conducted using an ACEA Novocyte 3000 cytometer. Data analysis was conducted in NovoExpress software.

**TABLE 1:** Antibodies used for flow cytometry.

Antibody	Fluorophore	Marker	Manufacturer	Cat no.	Clone
CD45	APC/Cyanine7	Immune cells	Biolegend	103 116	30‐F11
CD326 (EpCAM)	Brilliant Violet 421	Epithelial cells	Biolegend	118 225	G8.8
CD3	APC	T cells	Biolegend	100 236	17A2
CD4	APC	T cells	Biolegend	100 515	RM4‐5
CD8a	Brilliant Violet 421	T cells	Biolegend	100 737	53–6.7
CD19	Brilliant Violet 421	B cells	Biolegend	115 537	6D5
CD11b	Brilliant Violet 605	Myeloid cells	Biolegend	101 237	M1/70
F4/80	Brilliant Violet 785	Macrophages	Biolegend	123 141	BM8
ASGR1	–	Hepatocytes	BD	742 697	8D7
Fc block	–	–	Biolegend	101 320	93
7‐AAD	7‐AAD	Dead cells	Invitrogen	A1310	–
iNOS	APC	Macrophages	eBioscience	17–5920‐82	CXNFT
Arginase 1	eFluor 450	Macrophages	eBioscience	48–3697‐82	A1exF5
CD11b	PE	Myeloid cells	Biolegend	101 207	M1/70
F4/80	Brilliant Violet 421	Macrophages	Biolegend	123 131	BM8

Analyzing intestinal cell populations by flow cytometry, especially cells from mouse pups, presents unique challenges. Dramatic physiological changes occur in the mouse's digestive system during the first few weeks of life. At 7 days old, a mouse pup's diet consists entirely of milk, but by the time of weaning at 21 days, the pup eats a diet of solid food, and the intestines more closely resemble adult intestines (Figure S2A). These changes in diet and physiology affect the background fluorescence in flow cytometry studies (Figure S2B). Therefore, it is necessary to use nonfostered mice of the same ages as the fostered mice for control groups. Next, it is important to draw flow cytometry gates carefully. Intestines are complex and contain debris and dead cells, which are autofluorescent and easily mistaken for fluorescent milk cells. Therefore, we used a gating strategy that excludes autofluorescent cells and debris by plotting tdTomato against another unused fluorophore (Figure S3). Representative cell‐specific gating strategies for the lamina propria, mesenteric lymph nodes, blood, spleen, and thymus are shown in Figures S4–S8.

When quantifying a rare cell population by flow cytometry, noise from background fluorescence and nonspecific antibody staining presents complications. Therefore, careful controls and gating strategies are essential. Organs have varying levels of background fluorescence, so, for each organ, nonfostered C57BL/6 mice were used as fluorescence minus one (FMO) controls, and mT/mG mice were used as positive controls. Gates were drawn to include all the cells in the positive control mouse organs and exclude the cells in the FMO controls.

### 2.5 Imaging

Intestinal pieces were flushed with PBS and then flash frozen in liquid nitrogen. The frozen tissue was embedded in OCT and then cryosectioned in 7 μm slices. The slides were then washed with PBS to remove OCT, and tissue was stained with 1:500 Hoechst 33342 and 1:50 AF647‐phalloidin (Cell Signaling Technologies) for 1 h. Slides were imaged on a Zeiss 880 laser scanning confocal microscope. Nonfostered C57BL/6 mice were used as negative controls, and mT/mG mice were used as positive controls. Image analysis was conducted using ZEN software and ImageJ.

### 2.6 Extracellular vesicle isolation and characterization

Extracellular vesicles from milk were isolated by a combination of ultracentrifugation and size exclusion chromatography.^27^, ^28^, ^29^ First, mouse milk was centrifuged at 2000 × *g* for 10 min at 4°C to separate off the fat layer, which was then removed, yielding skim milk. Fifty microliters of this skim milk was then centrifuged at 10 000 × *g* for 30 min at 4°C. The supernatant was then ultracentrifuged at 100 000 × *g* for 3 h (TL‐100 benchtop ultracentrifuge, Beckman‐Coulter). The obtained crude EV pellet was washed in PBS once at 100 000 × *g* for 3 h. The washed pellet was resuspended in 1 mL of PBS, and EVs were purified by mini‐size exclusion chromatography (mini‐SEC) using 1.5 × 12 cm mini‐columns (Bio‐Rad, Hercules, CA, USA; Econo‐Pac columns) packed with 10 mL of Sepharose 2B (Millipore‐Sigma, St. Louis, MO).

Crude EVs (1.0 mL) obtained from ultracentrifugation were loaded onto the column, and five 1 mL fractions corresponding to the void volume peak were collected in PBS^27^ Fraction four was collected and used for subsequent experiments as the “EV” fraction. The fourth fraction was confirmed to contain milk EVs using three independent techniques as per MISEV2018 guidelines.^30^ (1) transmission electron microscopy (TEM) to observe classic EV‐like structures, (2) Western blotting to detect EV‐enriched markers (TSG101 and CD9), and (3) nanoparticle tracking analysis (NTA) to determine a size distribution profile with an average size of approximately 100 nm.^31^

The concentration and size distribution of EVs were measured by NTA using NanoSight 300 (Malvern, UK). First, the vesicles were diluted in ddH_2_O, and then the video image was captured at the camera level of 14. The captured videos were analyzed using NTA software, maintaining the screen gain and the detection threshold at 1 and 5, respectively. To determine mean particle size/concentration in each sample, three consecutive measurements were obtained and averaged. Given that SEC is a size‐dependent assay, we anticipate that the EVs obtained using this approach contain a heterogeneous mixture of exomeres, EVs, and microvesicles in the size range of 30–200 nm.^31^

### 2.7 On‐bead extracellular vesicle flow cytometry

EV membrane stability in the presence of simulated gastric fluid (SGF) was assessed using on‐bead flow cytometry as previously described.^32^, ^33^ Calcein Deep Red (AAT Bioquest Inc., Sunnyvale, CA) was solubilized in DMSO and diluted with 1× PBS to a final concentration of 10 μM. EVs were isolated and incubated with Calcein Deep Red prior to treatment with SGF for 30 min. The calcein reagent becomes fluorescent only within intact EVs.^32^ The SGF formulation used was modified to reflect infant stomach conditions.^34^, ^35^, ^36^ The buffer consisted of 34 mM sodium chloride and 19 mM potassium chloride, adjusted to pH 4 with hydrochloric acid and then spiked with 80 U/mL porcine pepsin (Sigma). Nontreated EVs and sonicated EVs were used as controls. CD63‐conjugated magnetic beads (ExoCap, MBL International, Woburn, MA) were prepared as previously described.^37^ Briefly, monoclonal anti‐CD63 antibody (MA5–24169, Invitrogen, Carlsbad, CA) was biotinylated using a one‐step antibody biotinylation kit purchased from Miltenyi Biotec (Auburn, CA) as recommended by the manufacturer. Biotinylated CD63 antibody (5 μg) was incubated with thoroughly washed 0.5 mL of streptavidin‐coated magnetic beads (1 × 108 beads/mL) for 1 h at 23°C under constant agitation. Ten micrograms of EVs (controls/SGF treated for 30 min at 37°C) were each incubated with 100 μL of CD63‐conjugated magnetic beads for 18 h (overnight) at 4°C under constant agitation. To assess membrane integrity, EVs were captured on CD63 magnetic beads, washed three times with PBS, and 100 000 events/group were assessed using NovoCyte 3000 flow cytometer (Agilent Technologies, Santa Clara, CA). Data were analyzed using NovoExpress software (Agilent Technologies, Santa Clara, CA).

### 2.8 Bone marrow‐derived macrophages

Bone marrow‐derived macrophages were isolated and cultured as previously described.^38^ Briefly, C57BL/6 mice of at least 8 weeks of age were euthanized via CO_2_ asphyxiation followed by cervical dislocation. The hindlimbs were disinfected with 70% isopropanol. The hindlimb skin was excised to expose the muscle. Tibiae and femurs were removed and cleaned of muscle. The bones were washed in media, and then both ends of each bone were removed using sterile scissors. Media was flushed through each bone to remove the cells. Cells were centrifuged and then resuspended in fresh media and then strained using a 70 μm filter. Cells were counted and then seeded in 96‐well plates at 200 000 cells per well. Bone marrow cells were cultured in DMEM/10% FBS/10% L929 conditioned media/1% Pen/Strep/2% NEAA/1% HEPES for 7 days, changing the media every 2 days.

To polarize macrophages to M1 (pro‐inflammatory) and M2 (anti‐inflammatory), macrophages were treated with 20 ng/mL IFN‐γ and 100 ng/mL LPS for M1 or 20 ng/mL IL‐4 M2 for 24 h. Following polarization, the media was replaced by normal media. Macrophages were treated with whole milk, milk cells, or milk EVs (all derived from mT/mG mice) for 24 h.

### 2.9 Macrophage flow cytometry

To characterize the uptake of milk cells or EVs, as well as the effect on macrophage polarization, macrophages were analyzed via flow cytometry following treatment. Macrophages were washed with PBS and then removed from the plates using Accutase. Following detachment, macrophages were transferred to round‐bottom plates and centrifuged. Cells were fixed using fixation buffer (R&D Biosystems) for 10 min and then quenched in flow buffer (5% FBS/1X PBS). Cells were Fc blocked using 1:200 anti‐mouse Fc Block (Biolegend) for 10 min. Macrophages were then incubated in antibody solution (1:100 Pacific Blue) anti‐arginase (Thermo Fisher), 1:100 APC anti‐iNOS (Thermo Fisher) in Permeabilization/Wash buffer (R&D Biosystems) for 45 min. Cells were washed in flow buffer and then run on a NovoCyte flow cytometer.

### 2.10 Statistics and data analysis

All statistical analysis was conducted using GraphPad Prism 8 software. A significant difference was defined as *p* < .05. All error bars on graphs show mean and standard deviation.

## 3 RESULTS

### 3.1 Milk cell components reach the intestines of fostered mice

To understand maternal milk cell fate in infant mice, we began by asking whether maternal milk cells and/or cell components travel through the stomach into the intestine, as cell uptake would most likely occur via the intestinal epithelium. Previously, we determined that only 27% of milk cells survive gastric digestion and reach the intestine intact.^39^ Only this subset of cells would subsequently be available for systemic uptake from the intestine. We considered that another possibility could derive from the 73% of cells that are digested in the stomach, as they would enter the small intestine in the form of cellular debris. Specifically, we hypothesized that digested milk cell components were entering the infant's systemic circulation, enabled by the combination of the infant's mild digestive conditions^40^ and the more permeable gut barrier.^41^, ^42^

**FIGURE 1:**
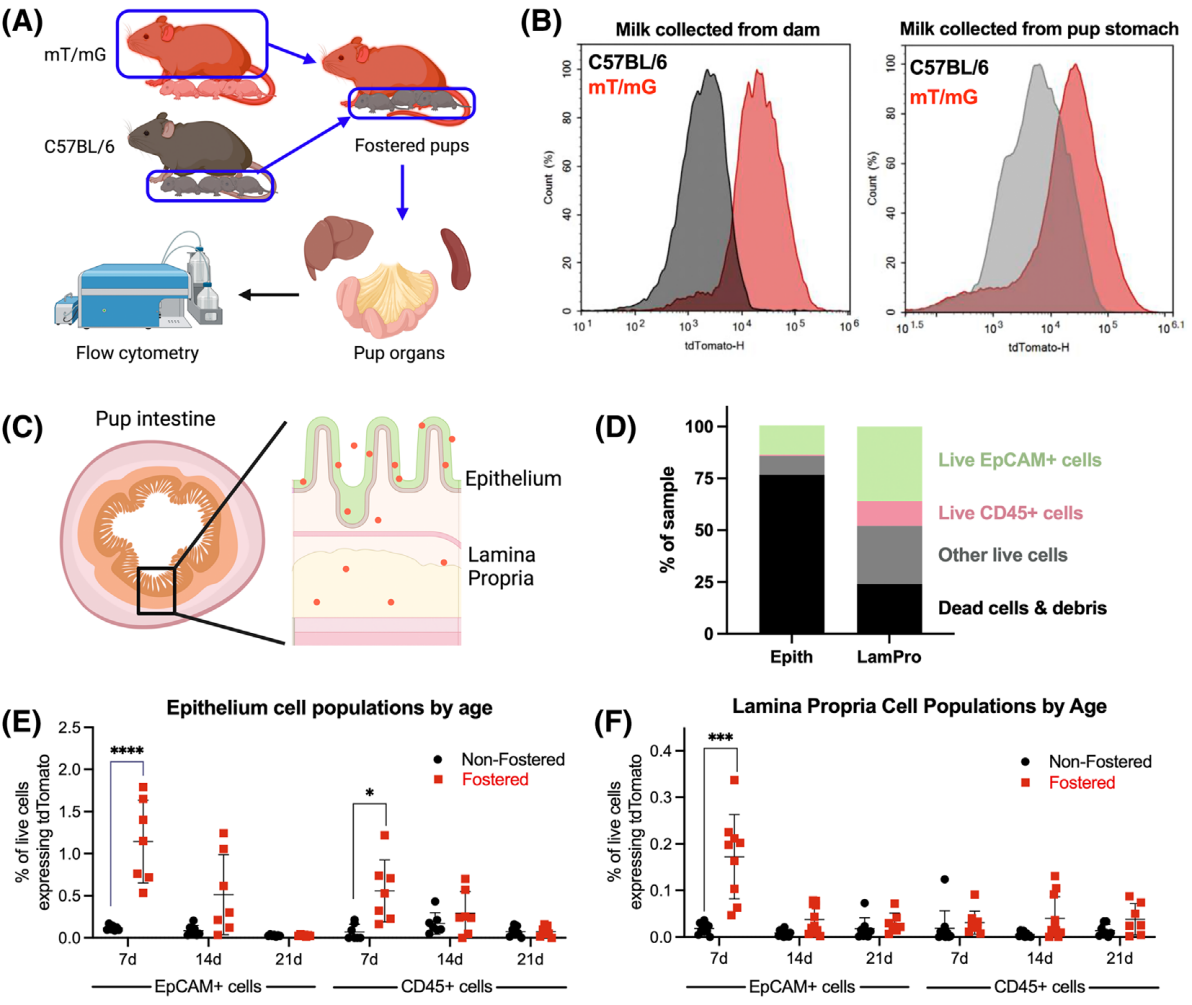
A foster nursing mouse model identified maternal milk cell components in pup intestines. (A) All experiments were conducted with an mT/mG foster nursing model. After wild‐type C57BL/6 mice (black) and mT/mG tdTomato+ (red) mice gave birth, their litters were swapped. Only the wild‐type pups were used in this study. Therefore, in wild‐type pups fostered by mT/mG dams, and any tdTomato signal was attributable to milk cell components. Negative control pups were nonfostered wild‐type mice. Organs were harvested from pups and analyzed by flow cytometry and/or microscopy for fluorescent signal. (B) Milk was collected directly from the dam and from pup stomachs and analyzed by flow cytometry. For both samples, mT/mG milk was confirmed positive for tdTomato signal. (C) To analyze milk cell transport into intestinal tissue, the epithelial intestinal layer and the lamina propria were dissected and analyzed by flow cytometry for tdTomato in 7, 14, and 21‐day‐old pups. Nonfostered wild‐type pups served as negative controls. Cells were stained for EpCAM (epithelial cells) and CD45 (immune cells). (D) The breakdown of cell types in the epithelium and lamina propria samples is shown. (E) For the epithelial layer, 7‐day‐old pups had increased tdTomato signal in both epithelial and immune cells. (F) For the lamina propria, 7‐day‐old pups demonstrated increased signal, but only for epithelial cells. Error bars represent mean ± SD. *p < .05, ***p < .001, ****p < .0001 by Student's *t*‐test, *n* = 8–10 for lamina propria and *n* = 7 for epithelium. Schematics created with BioRender.

To probe this hypothesis, we used a fluorescence‐based foster nursing model using mT/mG mice (Figure 1A). These mice have a C57BL/6 background and are genetically engineered to constitutively express the fluorescent protein tdTomato on the surface of every cell, including milk cells.^43^ For this model, wild‐type C57BL/6 and fluorescent mT/mG dams birthed litters on the same day, and their litters were swapped within 24 h of birth. We then examined only the fostered wild‐type pups, in which any fluorescent signal would derive from the mT/mG foster dam's milk cells. For negative controls, we used nonfostered wild‐type pups. Figure 1B shows that milk from mT/mG dams fluoresces compared to C57BL/6 milk, whether collected directly from the dam or from the pup's stomach.

In the intestines of the mouse pups, we considered the intestinal epithelium and lamina propria separately (Figure 1C), given that any signal in the lamina propria would have traversed the epithelial barrier. The most abundant cell type in both tissue types was epithelial cells (Figure 1D). Cells from the epithelium were less viable than from the lamina propria, as the tissue is quite delicate. We analyzed cell populations for tdTomato, comparing C57BL/6‐fostered mouse pups to age‐matched nonfostered C57BL/6 mouse pups. In the intestinal epithelium, fluorescent epithelial cells accounted for ~1% of all cells in 7‐day‐old fostered mice and ~0.5% in 14‐day‐old fostered mice (Figure 1E). Fluorescent CD45+ cells (immune cells) were also present in the epithelium for only 7‐day‐old mice. In the lamina propria, ~0.2% of live cells expressed tdTomato in 7‐day‐old fostered mice but not in older mice (Figure 1F). No fluorescent signal was detected in immune cells. These data showing a decline in tdTomato with age are consistent with pup feeding patterns. At 7 days, their diet is exclusively milk, and signal is the highest. By 14 days, pups have begun transitioning to solid food, and by 21 days, the pups no longer consume milk.

**FIGURE 2:**
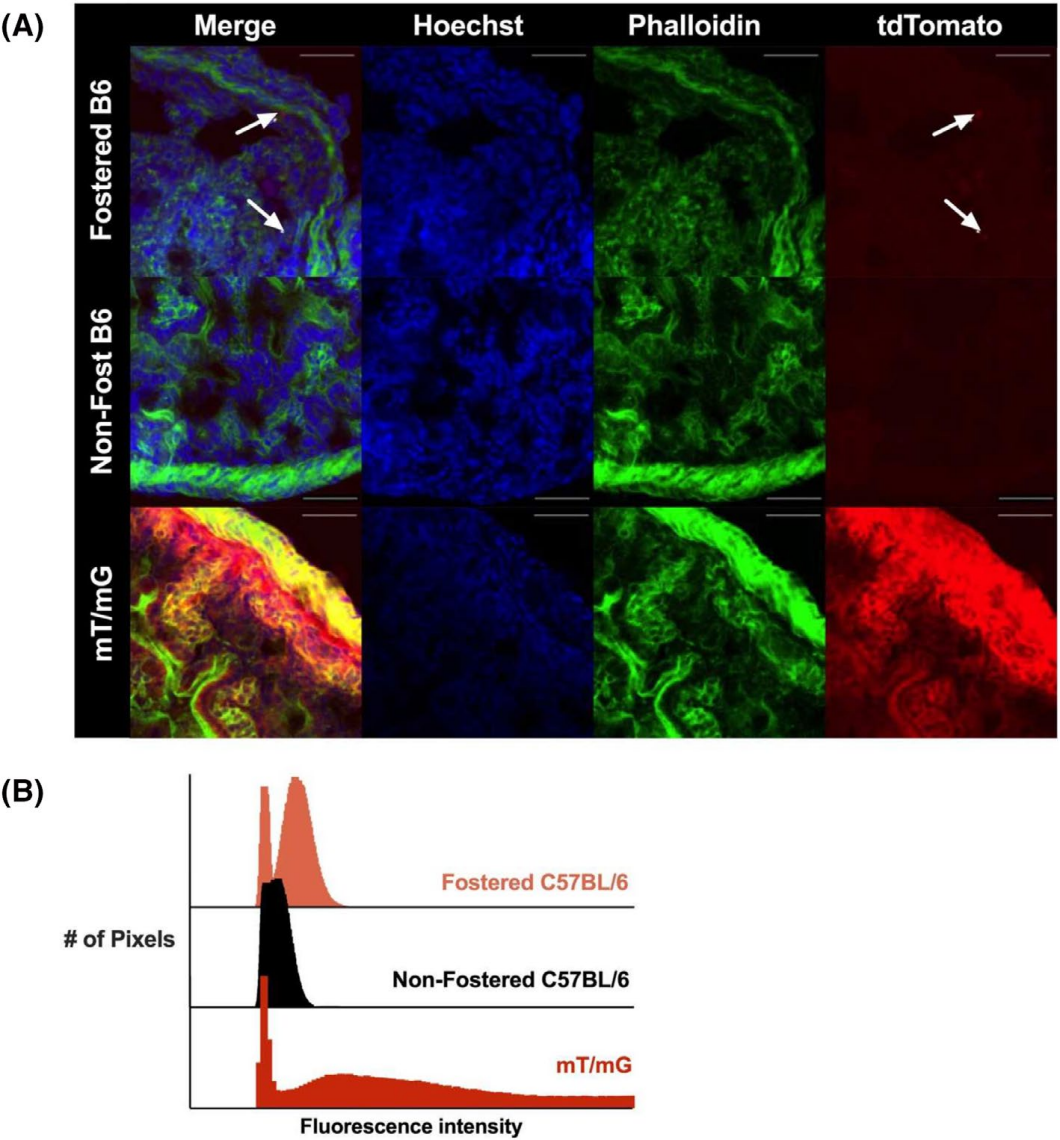
Confocal image analysis identified milk‐derived fluorescent signal in the intestines of fostered mice. (A) Confocal imaging was performed on the intestines of 7‐day‐old mice. Intestinal sections were flash frozen and stained with Hoechst (blue) and phalloidin (green). TdTomato signal in the fostered C57BL/6 (B6) mice is indicated by white arrows. TdTomato signal was absent in the wild‐type C57BL/6 mice (negative control) and expressed constitutively in mT/mG mice (positive control). Scale bars: 50 μm. (B) In the confocal images for each group, tdTomato intensity was quantified for all pixels. Images of fostered mouse intestines have higher levels of tdTomato fluorescence compared to nonfostered mice but lower than positive control mice.

In addition to flow cytometry, we used confocal imaging to detect tdTomato in the intestines of 7‐day‐old mouse pups. Intestinal sections were stained with Hoechst and phalloidin to label cell nuclei and F‐actin architecture, respectively (Figure 2A). The tissue of mT/mG mouse pups robustly expressed tdTomato (positive control), whereas the nonfostered C57BL/6 pups had only background signal (negative control). In fostered C57BL/6 pups, we identified faint spots of tdTomato throughout the intestinal tissue. Although the tdTomato signal can be difficult to visualize in individual images, a histogram of all the pixels in the confocal images confirmed the increase in tdTomato signal for fostered pups compared to nonfostered pups (Figure 2B).

Regarding our hypothesis that digested milk cell components were entering infantile tissue, we considered that tdTomato is a membrane‐bound reporter protein. Therefore, any intact cells transferred from mother to infant should express tdTomato around the cell perimeter.^43^, ^44^ This is illustrated in the mT/mG positive control samples (Figure 2A), which show the expected pattern of tdTomato around the outline of the cells. Looking closely at the confocal images from the fostered pups, however, tdTomato appeared as small spots within intestinal cells of the fostered pups (Figure S9). This suggested that tdTomato signal observed in mouse pups may not be solely attributable to the transport of intact milk cells into the pup but instead due, at least in part, to the uptake of digested milk cell fragments into phagocytic pup cells.

### 3.2 Milk cell components were not detected in the organs of the immune system

Next, we asked whether maternal milk cells and/or cell components were accumulating in pup tissue beyond the intestine. We were interested in accumulation within organs of the immune system, given their important role in the response to foreign materials. Indeed, previous work has reported the presence of milk‐derived immune cells in the infant mesenteric lymph nodes,^20^ spleen, and thymus.^22^ We sought to confirm these results, expand these data to all types of maternal milk cells, and include the blood as an additional component of the immune system.

**FIGURE 3:**
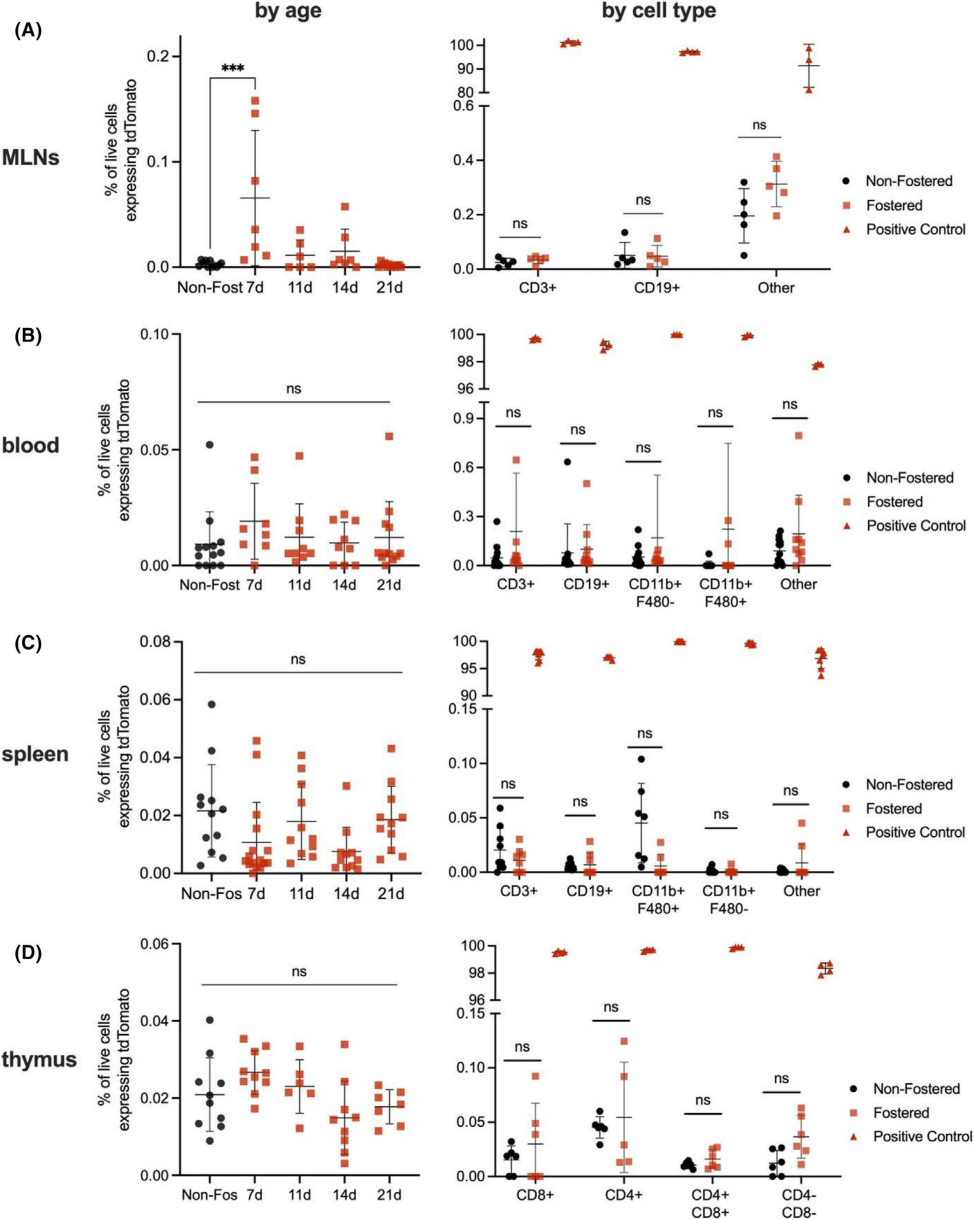
Only the lymph nodes of 7‐day‐old fostered mice contained milk‐derived tdTomato. Here, tissue samples from fostered mice 7–21 days old were examined by flow cytometry for tdTomato+ cells. In the left panels, fostered mice were compared to negative control nonfostered pups. The samples from 7‐day‐old mice were then analyzed for tissue‐specific cell types (right panels). Nonfostered C57BL/6 and tdTomato‐expressing mT/mG pups served as negative and positive controls, respectively. (A) Only mesenteric lymph nodes contained significant tdTomato signal, and only in 7‐day‐old fostered mice. (B) No tdTomato signal was visible in the blood, (C) spleen, or (D) thymus of fostered mice. *n* = 6–15 for age groups, *n* = 6–12 for cell types. Error bars represent mean ± SD, ***p < .001 by one‐way ANOVA with post hoc comparison to the nonfostered group. Cell type statistics calculated by Student's *t*‐test.

For these experiments, we used flow cytometry to analyze cell populations from the mesenteric lymph nodes, blood, spleen, and thymus of fostered mouse pups up to 21 days of age. We detected an increase in tdTomato+ cells in the lymph nodes of 7‐day‐old fostered mice (Figure 3A, left), but this increase above background was present in only a few of the mice. Most cells in the lymph nodes are T and B cells, which we identified with the markers CD3 and CD19, respectively.^45^ Comparing the T cell and B cell populations of fostered and nonfostered mice, we did not identify a population of tdTomato+ cells in either cell type (Figure 3A, right). Additionally, there was a nonstatistically significant increase in the group of “other” immune cells (CD45+ CD3‐ CD19‐) in the mesenteric lymph nodes. Increased signal is likely due to dendritic cells, given their role in presenting antigens to lymphocytes.^46^, ^47^ Positive control mT/mG pups expressed tdTomato in nearly 100% of cells.

Beyond the mesenteric lymph nodes, milk‐derived cells were absent from the blood (Figure 3B), the spleen (Figure 3C), and the thymus (Figure 3D) of fostered mice. From these results, we concluded that neither whole milk cells nor milk cell fragments (free‐floating or phagocytosed) were accumulating in organs of the immune system. Therefore, we examined other potential mechanisms for milk cell and/or milk cell component transport out of the intestine.

### 3.3 TdTomato was detected in the Kupffer cells of the livers of fostered mice

As an alternative to milk cell uptake through immune organs, we considered that milk cell and/or cell components could travel from the intestine to the liver via the hepatic portal circulation. Although the liver is not traditionally considered an immune organ, it is essential to the body's immune defenses, especially in removing foreign material from the blood coming from the intestines.^48^ There is precedence for milk cell uptake in the neonatal liver in a 1989 study in baboons^8^ and a 2000 study in mice.^19^ However, these studies did not include characterization of cell types. Separately, Ghosh and coworkers identified milk‐derived cells in the livers of fostered mice but focused only on T cells.^22^ Therefore, we analyzed pup livers for all tdTomato+ cells and characterized them by cell type.

**FIGURE 4:**
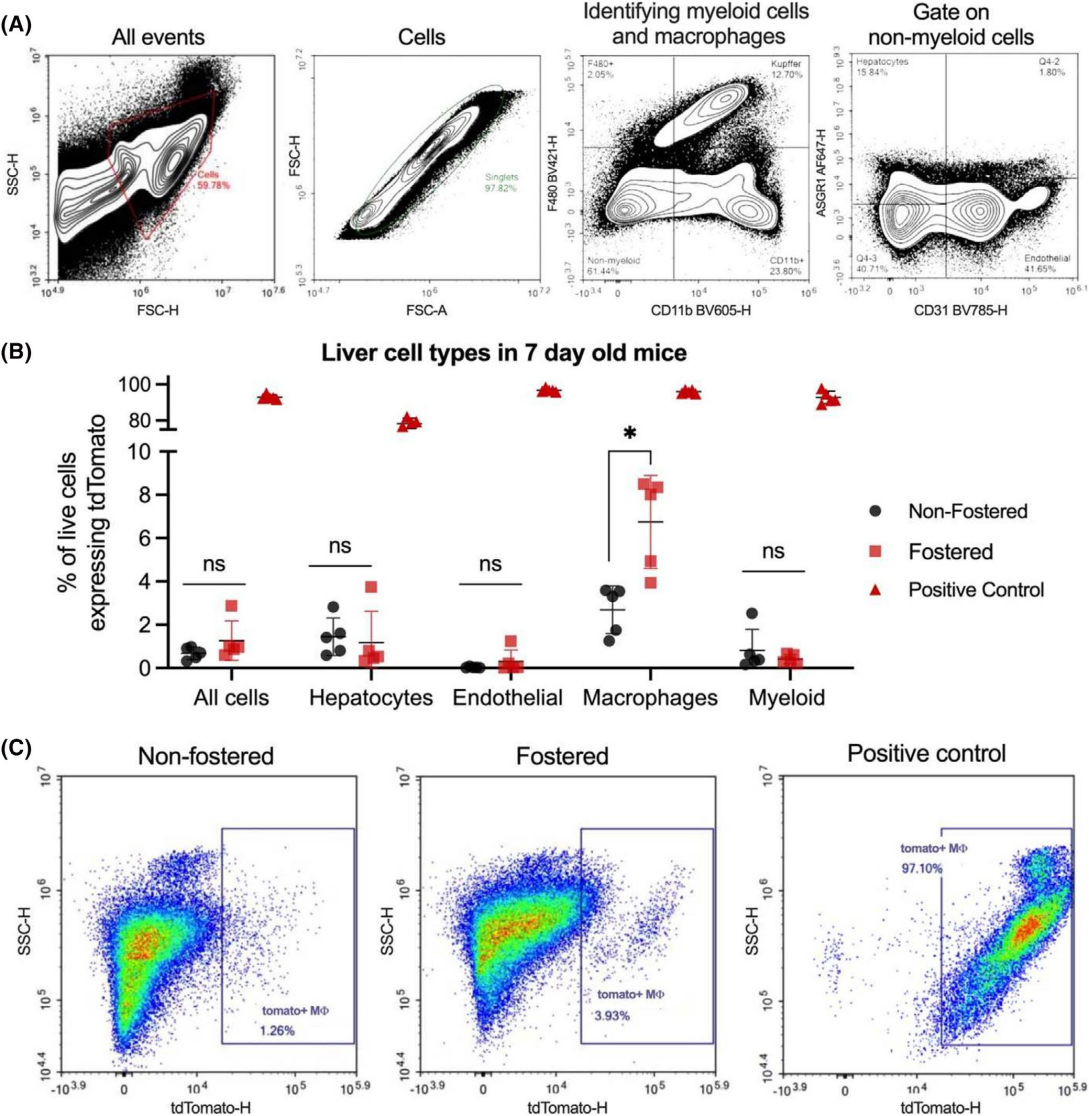
Liver macrophages from 7‐day‐old pups contained milk‐derived tdTomato signal. Fostered mice, nonfostered C57BL/6 mice (negative control), and mT/mG mice (positive control) pups were sacrificed seven days postbirth, and their livers were analyzed by flow cytometry. (A) Hepatocytes were defined as CD11b‐ CD31‐ ASGR1+, endothelial cells were defined as CD11b‐ ASGR1‐ CD31+, macrophages were defined as CD11b + F4/80+, and myeloid cells were defined as CD11b + F4/80‐. (B) Within the livers of fostered mice, macrophages were the only cell type containing tdTomato signal compared to wild‐type C57BL/6 mice. (C) Gates to identify tdTomato+ macrophages were drawn around the population of macrophages from positive control mice; the nonfostered control group shows some noise, but the fostered group has a well‐defined population of positive cells. *n* = 5 mice per group, error bars represent mean ± SD, *p < .05 by Student's *t*‐test.

For these experiments, we used flow cytometry to characterize several liver cell populations in 7‐day‐old fostered mice: hepatocytes, endothelial cells, macrophages, and myeloid cells (Figure 4A). The only population with a significant increase in tdTomato expression was the CD11b + F4/80+ cells, of which approximately 5% were tdTomato+ when subtracting off the negative control background signal (Figure 4B). These cells could predominantly be Kupffer cells (liver‐resident macrophages) that are active in mouse pups as young as 3 days or maternal macrophages that have traveled from the pup intestine to the liver.^49^ Kupffer cells phagocytose foreign material from the intestine to prevent it from reaching the rest of the body, and they have been described as the “final component in gut barrier function.”^50^ On the flow cytometry plots, the tdTomato+ CD11b + F4/80+ cells appeared as a distinct population (Figure 4C). However, quantification of only the macrophage markers CD11b and F4/80 could not determine whether these cells were maternal milk macrophages that had migrated to the liver or infant macrophages that had phagocytosed fluorescent, cell‐membrane‐derived components from the milk.

### 3.4 Origin of tdTomato+ cells in the pup

To further elucidate the mechanism of tdTomato accumulation in the infant, we stepped back to consider all analyzed tissues. Fluorescent signal was observed in intestinal epithelial cells of both the epithelial and lamina propria layers and in intestinal CD45+ immune cells in the epithelial layer as well as in CD11b + F4/80+ liver cells (i.e., macrophages). To determine whether these cells were maternal or infant in origin, we used flow cytometry to probe for these cell populations in milk. As shown in Figure S10, we found that milk contained an abundance of epithelial cells (~35%) but only miniscule numbers of monocytes and macrophages (~0.1%). This indicates that tdTomato+ epithelial cells in the pup intestine could be maternal milk cells, and it is also possible that these are pup epithelial cells that have taken up milk cell components.

Regarding the tdTomato+ liver macrophage cells in the pup, however, it is unlikely that they are maternal milk cells. Instead, it is likely that these are either pup Kupffer cells or pup intestinal macrophages that migrated to the liver, with the former being much more likely given that 5% of all liver macrophages expressed tdTomato (Figure 4B). In either case, the tdTomato signal in these pup cells would result from the uptake of milk cell components. Further, this uptake could have happened directly or indirectly. Direct uptake would occur through free‐floating milk cell components being taken up by macrophages, whereas indirect uptake could result from the transfer of extracellular vesicles containing milk cell components.

### 3.5 Extracellular vesicles were unaffected by gastric digestion compared to milk cells

Next, we considered what types of milk cell components were responsible for tdTomato signal in pup cells. Some options we considered included “primary” components: apoptotic milk cells, milk cell fragments, and milk extracellular vesicles (EVs), as well as “secondary” EVs secreted by pup immune cells that had phagocytosed and repackaged primary cell components.

We were especially interested in the potential role of EVs, which are cell‐derived vesicles that transfer cellular cargo, such as proteins and RNA, to other cells as a form of communication.^51^ The possibility of EV involvement is supported by prior research showing that EVs within the gastrointestinal tract interface with the immune system and are taken up by intestinal epithelial cells.^52^, ^53^ Further, our group and others have shown that phagocytic cells produce EVs containing phagocytosed materials that can then be transferred to other cells.^54^, ^55^, ^56^

**FIGURE 5:**
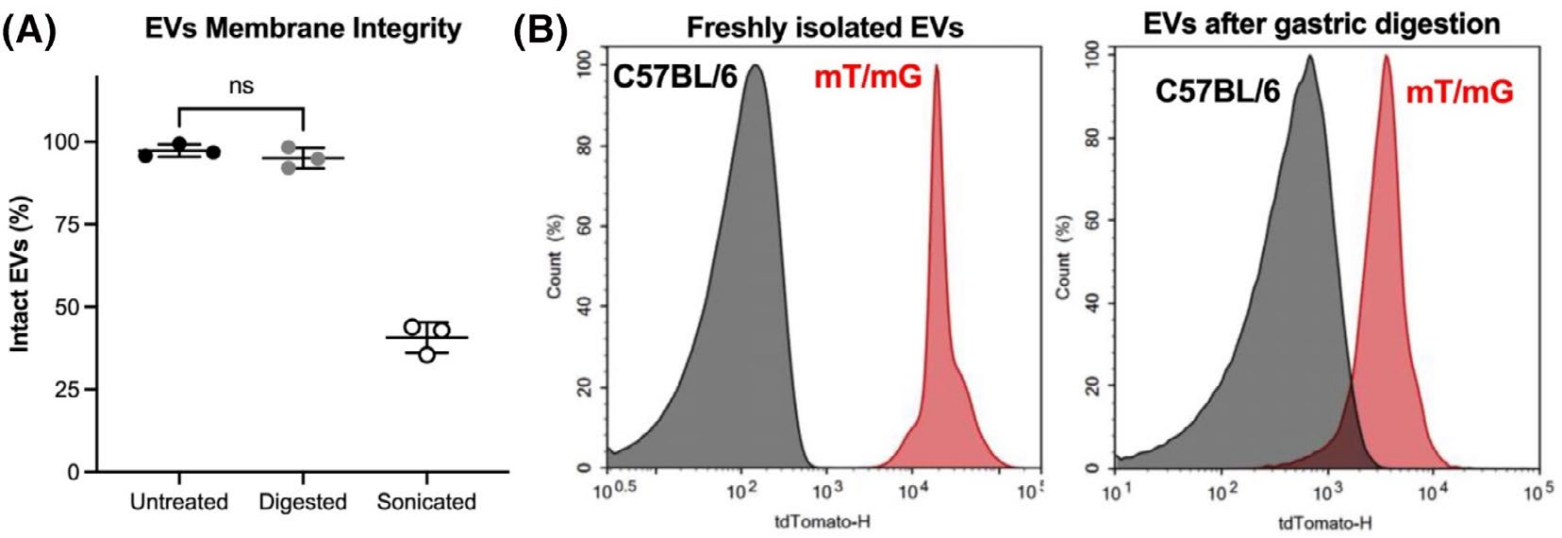
Milk extracellular vesicles maintained integrity and fluorescence upon digestion. EVs were isolated from freshly expressed mouse milk. (A) EVs did not lose their integrity postdigestion, as determined by on‐bead flow cytometry, with sonication‐induced disruption serving as a positive control. *n* = 3 mice, nonsignificant by Student's *t*‐test. (B) Flow cytometry determined that EVs isolated from mT/mG mouse milk (red) expressed tdTomato relative to C57BL/6 mice (black). EVs from mT/mG mouse milk retained a lower level of tdTomato fluorescence after 30 min of digestion in simulated gastric fluid.

To examine the possibility that milk‐derived EVs were responsible for tdTomato signal in the pup, we first characterized their properties before and after digestion. Specifically, we confirmed the presence of small EVs (50–250 nm) in mouse milk (Figure S11) as well as their ability to withstand digestion (Figure 5A). For digestion experiments, EVs isolated from freshly expressed milk were compared to EVs subject to simulated gastric digestion.^35^ Most EVs remained intact postdigestion (~95%), whereas sonication‐based membrane disruption (positive control) destroyed the majority of EVs. We also confirmed that EVs derived from the milk of mT/mG dams were tdTomato+ compared to wild‐type dams and retained their fluorescence postdigestion (Figure 5B). Together, these experiments confirm that tdTomato signal in the pup could derive from milk EVs, given that they maintain their integrity and fluorescence as they travel from the mother into the infant's intestines.

### 3.6 Macrophages phagocytose milk EVs and milk cells ex vivo

Given that tdTomato signal was detected in the macrophages of pup livers (Figure 4), we next confirmed that macrophages can take up tdTomato+ milk components. For these experiments, primary immune cells from C57BL/6 (wildtype) were differentiated into M0, M1, and M2 macrophage phenotypes and dendritic cells (DCs). The evaluation of macrophages in both M1 (pro‐inflammatory) and M2 (alternative) states is important, as their behavior and role in the body are considerably different.^50^ Once cells were differentiated, we incubated them with mT/mG milk cells or EVs before assessing uptake by flow cytometry. After 24 h, the cell populations were evaluated by flow cytometry for tdTomato fluorescence and macrophage polarization markers.

**FIGURE 6:**
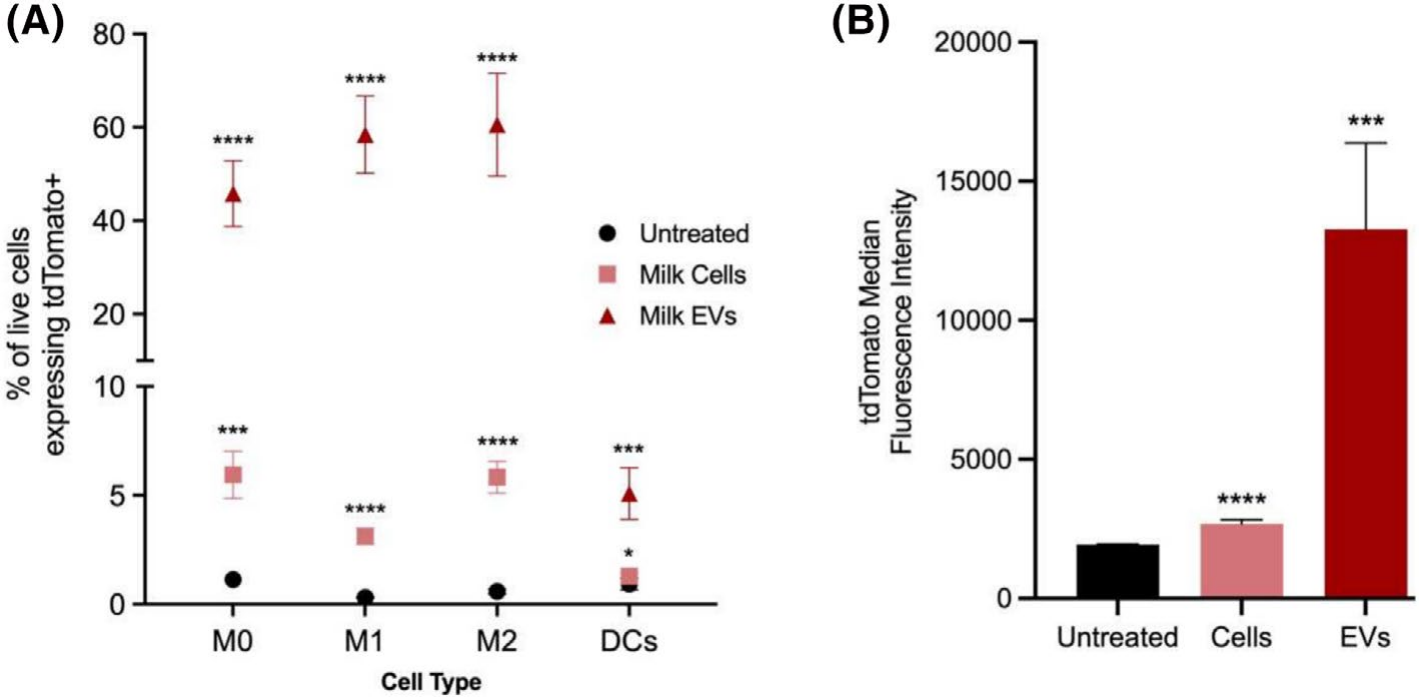
Macrophages took up milk EVs more readily than milk cells. Primary macrophages and dendritic cells were isolated from mice and cultured ex vivo. Macrophages were then differentiated into M0, M1, or M2 phenotypes. Milk was obtained from mT/mG (tdTomato+) mice, and the milk cells and milk EVs were isolated prior to their incubation with the primary cell types. (A) A greater percentage of macrophages and DCs took up EVs compared to milk cells. Untreated cells were used as a negative control. (B) Considering all macrophages together, they took up EV‐derived tdTomato to a greater degree than milk cell‐derived tdTomato. *n* = 4, error bars represent mean ± SD. *p < .05, ***p < .001, ****p < .0001 by Student's *t*‐test.

We found that a statistically significant number of each type of immune cell contained tdTomato after treatment with cells or EVs compared to untreated cells (Figure 6A). EV exposure resulted in the highest number of tdTomato+ cells: 40%–60% of each type of macrophage and 5% of dendritic cells. This was in contrast to milk cells, which resulted in only ~5% of each cell type to register as tdTomato+. For each primary cell type, we also calculated the mean fluorescent intensity following exposure to milk cells or milk EVs (Figure 6B), which shows that EV‐treated primary cells took up ~10× more tdTomato compared to milk cell‐treated primary cells. Regarding milk cell treatments, these experiments cannot conclusively determine whether tdTomato signal derived from uptake of whole cells or uptake of cell components (e.g., EVs, apoptotic bodies) that were generated during the incubation process.^57^ In either case, these data show that macrophages readily consume milk EVs and that, therefore, there is a reasonable chance that tdTomato signal in pup liver macrophages derives from EVs.

## 4 DISCUSSION

Previously published work suggested that live milk cells cross the intestinal barrier and integrate into the infant's body, particularly in the infant's immune organs.^18^, ^20^, ^21^, ^22^ Our results agree in that we detected maternal cell‐derived fluorescent signal in 7‐day‐old fostered mouse pups; however, our data suggest that systemic fluorescent signal is more likely attributable to the transintestinal transport of milk cell components than intact milk cells.

New advances in molecular biology and imaging, as well as new discoveries in basic science, have made it possible to collect data that was not available in the 1980s when the original studies on milk cell transfer were published. We now have high‐quality antibodies, multicolor flow cytometers, and confocal microscopes that enable the collection of detailed characterization data. In the original studies, foster nursing models were not possible because the fluorescent reporter animals did not exist—mice were first genetically modified in 1981^58^ the first fluorescent reporter mouse was developed in 1997^59^, ^60^ and the reporter mouse model used here was developed in 2007.^43^ In addition to these new methods, new fundamental discoveries were made, including the detection of EVs in human milk in 2007.^52^ Therefore, the authors initially reporting on the transfer of milk components from mother to infant would not have considered EVs as a potential explanation for their observation. Over the last decade, a growing body of literature has shown that EVs are involved in cell‐to‐cell communication by carrying complex cargo ranging from nucleic acids to proteins.^61^ It is likely that milk EVs have exciting properties in the infant that have not yet been described.

In our experiments, tdTomato+ signal was detected in intestinal epithelial cells and in the Kupffer cells of the liver. Additionally, we detected an increase in tdTomato+ cells in a population of CD45+ CD3‐ CD19‐ cells in the mesenteric lymph nodes, although this increase was not statistically significant due to our inability to collect enough lymph nodes to obtain adequate cell numbers. Literature suggests that these MLN cells are dendritic cells that sampled antigens in the intestine.^62^ After being taken up through the mesenteric lymph nodes, it is plausible that digested milk cell components and extracellular vesicles were taken via the hepatic portal circulation to the liver,^63^ where they were phagocytosed by Kupffer cells. One potential limitation of the present study is that the effects of cross‐fostering are not captured in the control groups. It is possible that pups could mount an immune response when exposed to milk antigens from a nonbiological dam, which could increase stress and alter the gastrointestinal environment. However, we anticipate any such effects to be small, given the healthy growth of fostered pups compared to nonfostered pups (Figure S1).

Our work suggests that EVs play an important role in the accumulation of tdTomato signal in liver cells. This is consistent with previous work showing that milk‐derived EVs cross biological barriers such as the intestinal barrier.^64^ To our knowledge, no study has simultaneously considered the transport of cells and EVs, which may be necessary to avoid confounding data. Based on literature, we expected that viable milk cells crossed the infant's intestinal barrier, and we sought to quantify the cell types that could cross the intestinal barrier and the kinetics of this transport process. However, we instead found a complex picture of numerous milk components interacting with the infant's digestive system and immune system.

While we cannot entirely rule out the possibility that maternal cells cross the infant intestinal barrier, it is likely that a more commonplace phenomenon is at play. Specifically, milk proteins derived from either extracellular vesicles or digested cells are being taken up by the infant's own cells, primarily by the infant's intestinal epithelial cells and macrophages. These infant cells resemble milk‐derived cells in the context of fluorescence‐based analysis.

The characterization of cells from milk is a new and rapidly evolving field, and new ideas are emerging to explain the fate of the milk cells. Early studies on milk cells often assumed that all cells in milk were immune cells^6^, ^8^ and therefore used immunology terms to describe their observations. Newer data from human milk shows that immune cells are only a small minority of the cells in milk,^65^, ^66^, ^67^ and therefore we can consider other possible explanations involving cell types beyond immune cells. In this research, we have built upon existing literature, reinterpreted previous reports, and developed a more complete understanding of the uptake of maternal milk cell components in the infant.

## AUTHOR CONTRIBUTIONS

Rose Doerfler, Saigopalakrishna Yerneni, Jilian R. Melamed, and Kathryn A. Whitehead conceived of and designed the research; Rose Doerfler, Saigopalakrishna Yerneni, Samuel LoPresti, Namit Chaudhary, Alexandra Newby, Jilian R. Melamed, and Angela Malaney performed the research; Rose Doerfler, Saigopalakrishna Yerneni, Samuel LoPresti, Namit Chaudhary, Alexandra Newby, and Jilian R. Melamed analyzed the data; Rose Doerfler and Kathryn A. Whitehead created the figures; Rose Doerfler and Kathryn A. Whitehead wrote the paper; all authors approved the submitted version.

## DISCLOSURES

The authors have no competing interests to declare.

## Supporting information


Data S1.

## Data Availability

The data generated for this study, including figures and datasets, are openly available at https://doi.org/10.1184/R1/28143575.v1.
