# Supplementary material for: Maternal milk cell components are uptaken by infant liver macrophages via extracellular vesicle mediated transport
Source: FASEB J. 2025 Jan 21;39(2):e70340. doi: 10.1096/fj.202402365R (PMC11748825; doi:10.1096/fj.202402365R)
Supplement: Supplementary file 1 — Data S1. [file FSB2-39-e70340-s001.pdf]

Supplementary Figures

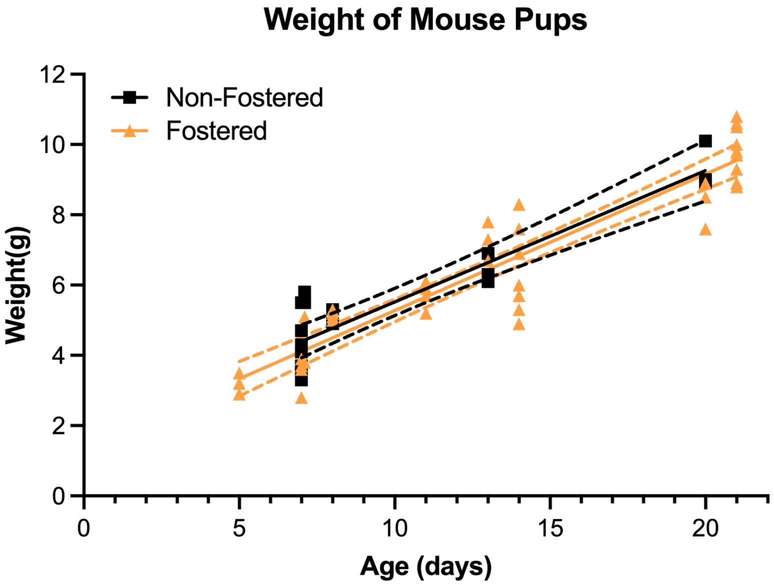

Figure S1. **Non-fostered and fostered pups gained weight at the same rate.** This demonstrated that fostered pups were accepted by their foster dam. N = 9 for non-fostered and fostered pups.

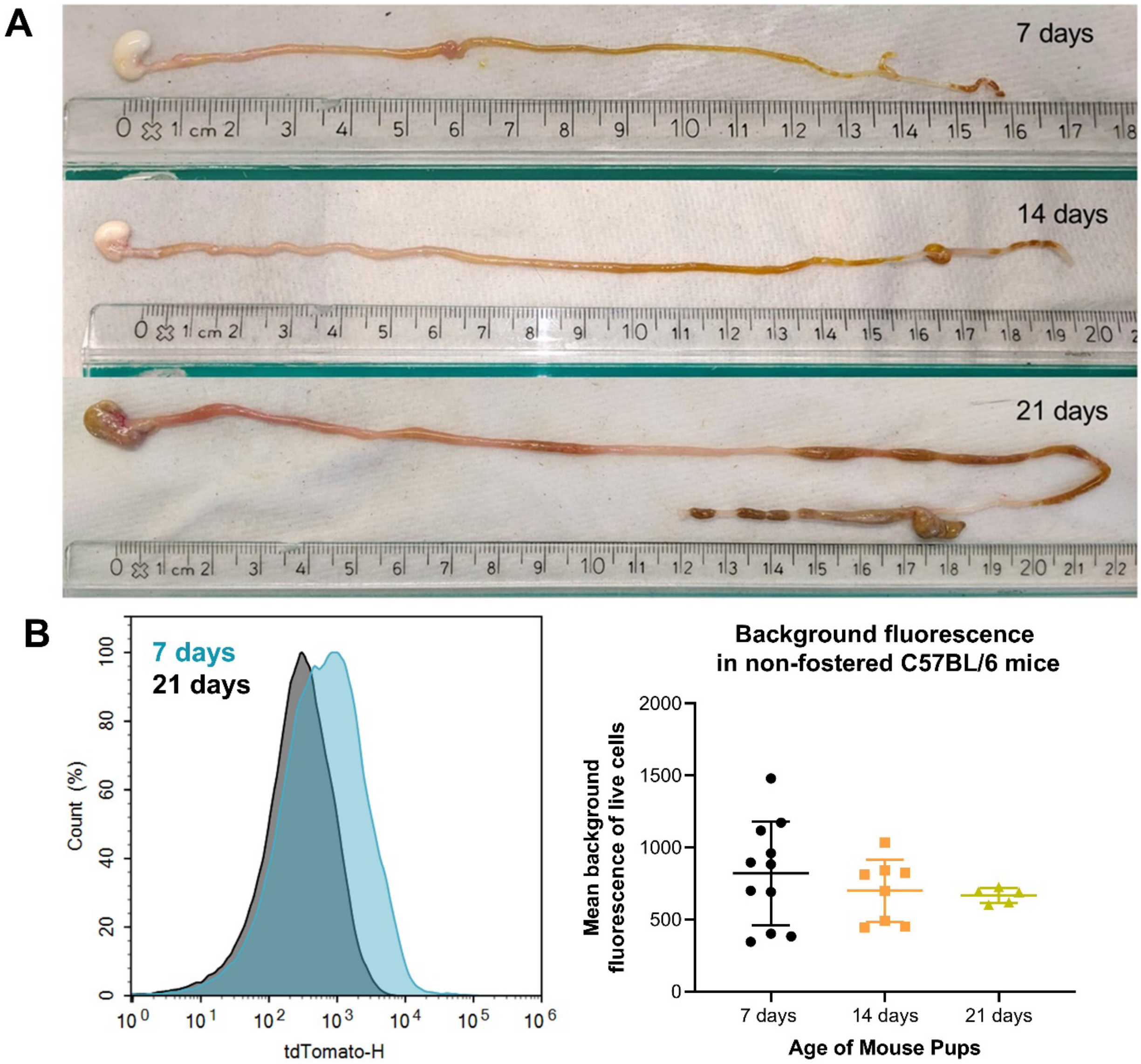

Figure S2. The physiology of mouse intestines changed dramatically during the first three weeks of life. Mice progress from a diet of only milk to a diet of only solid food during the first few weeks of life, and the changes in the appearance and physiology of the intestines during this time affect the background fluorescence in flow cytometry experiments. **A)** Before mice began to eat solid food, their stomachs were white with milk and their intestines appeared yellow. At the time of weaning, intestines appeared brown. **B)** The intestines of 7-day old mice had a higher tdTomato background fluorescence than 21-day old mice. Therefore, when analyzing intestinal flow cytometry data for young mouse pups, it is important to use mice of the same age as controls.

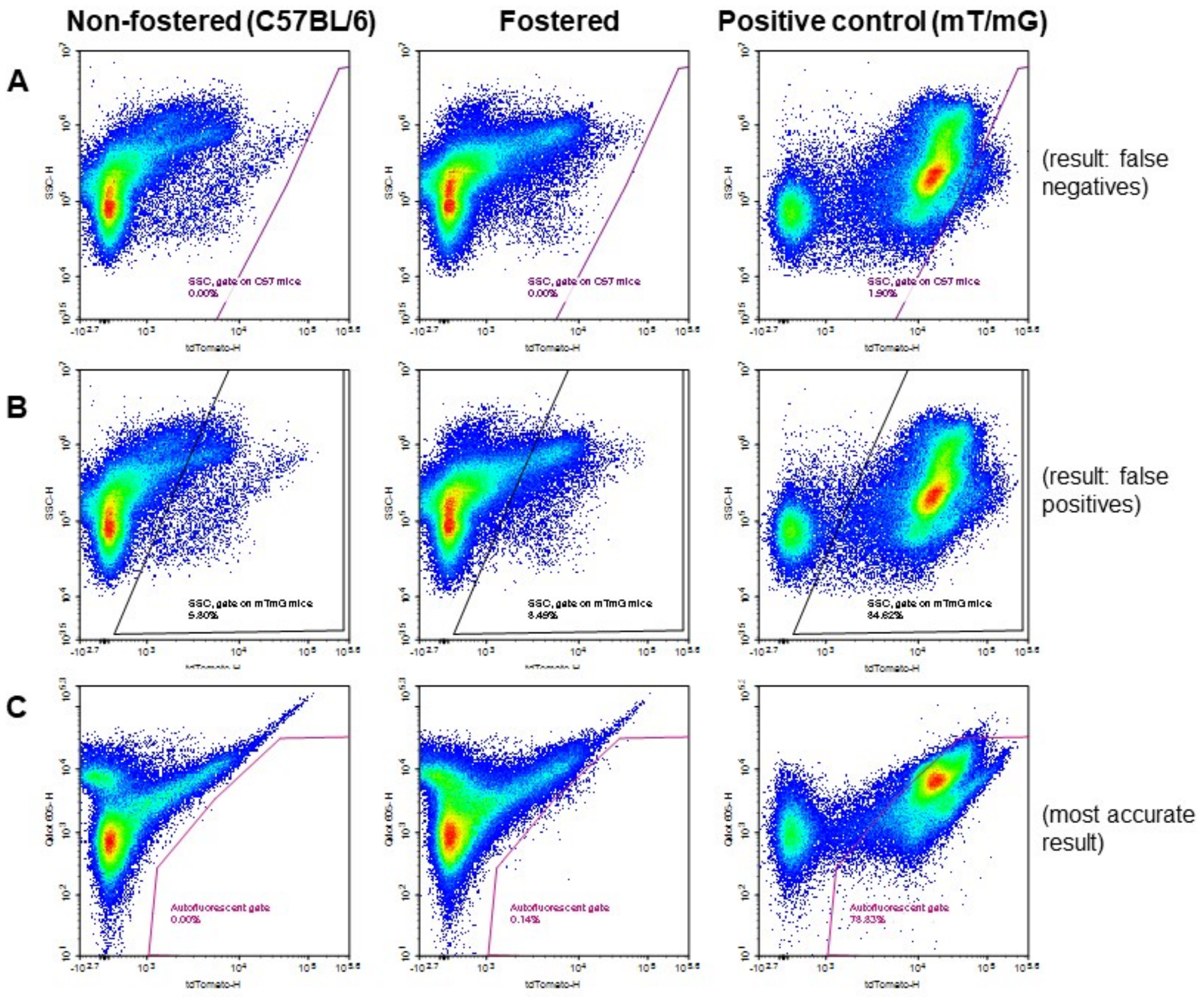

Figure S3. Strategy to gate out auto-fluorescent cells and debris in the intestine. A) Using a scatter plot of tdTomato against side scatter, it is impossible to draw a gate that includes all of the true tdTomato+ cells while excluding debris and noise. The typical strategy of drawing the gate to the right of the fluorescence minus one (FMO) control sample (i.e., the non-fostered C57BL/6 mice) yields false negative signal in the mT/mG mice. **(B)** Similarly, drawing a gate around the positive control samples (here, mT/mG mice) yields false positives in the non-fostered C57BL/6 samples. In this case, the non-fostered mouse intestine appears to include 5% tdTomato+ cells, which is impossible. **(C)** A better strategy is to plot tdTomato against another fluorescence channel that is not used in the panel. This strategy allows the drawing a gate that excludes events in the C57BL/6 non-fostered control mice while including the cells from the mT/mG positive control. Therefore, the resulting gate counts only true fluorescent cells and not debris, minimizing both false positives and false negatives.

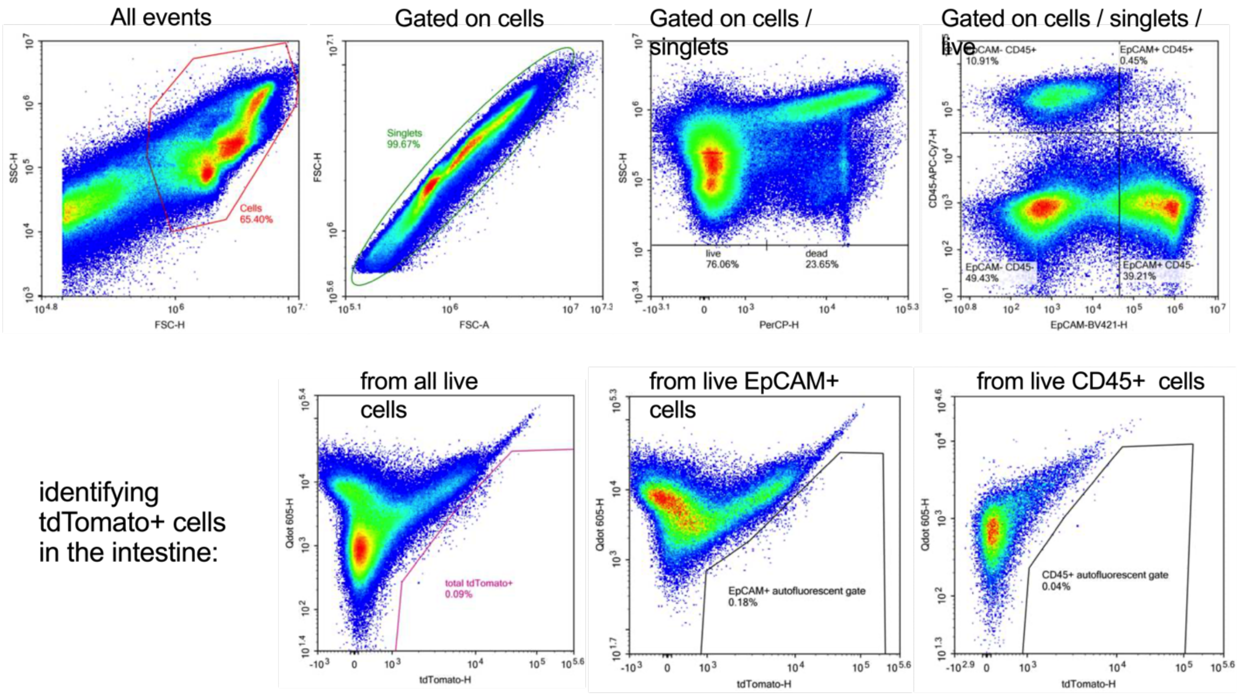

Figure S4. Gating scheme to identify cell type-specific TdTomato+ cells in the lamina propria. This is an example of the gating scheme used in Figure S3, in this case for cells of the lamina propria.

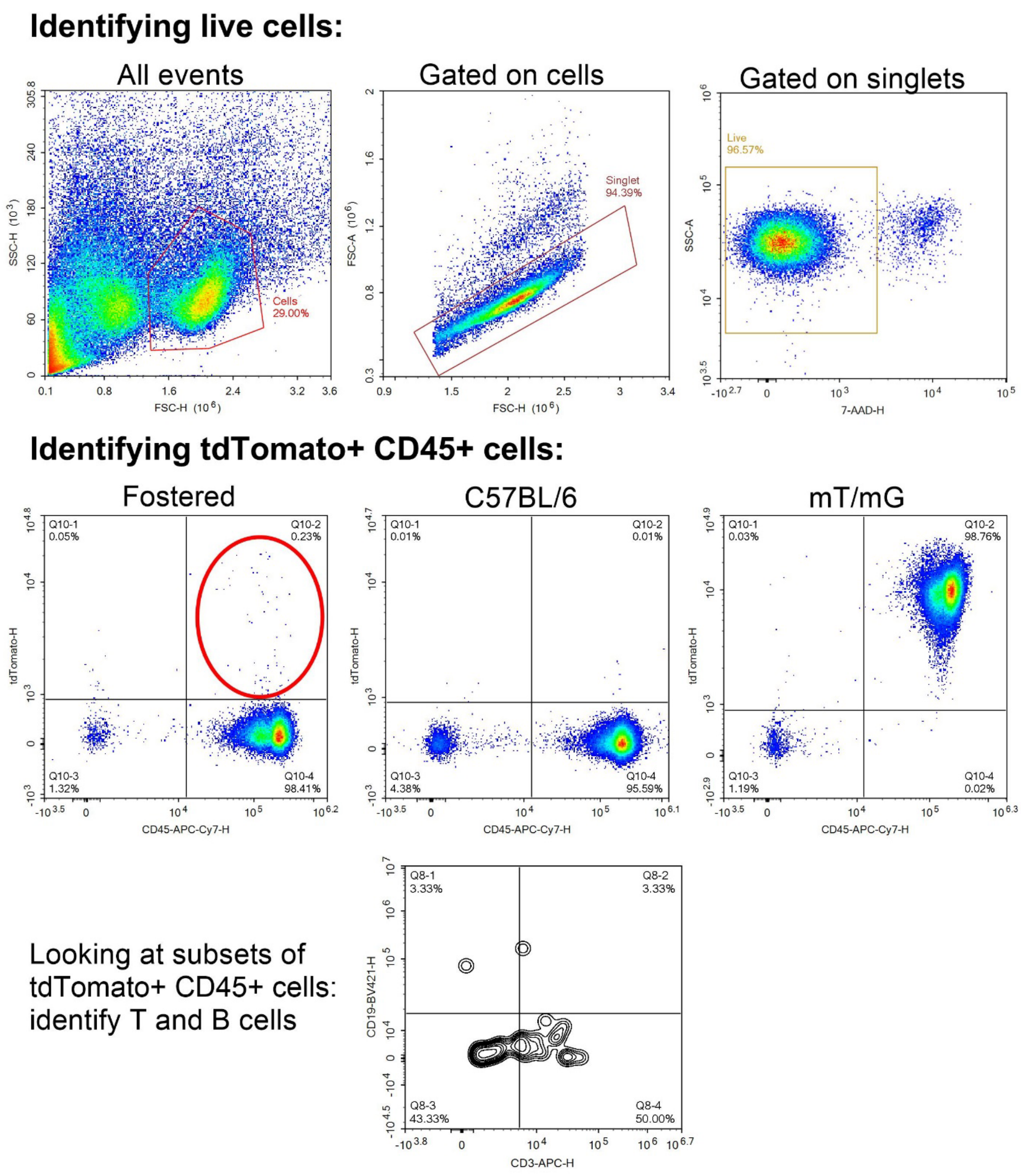

Figure S5. Gating scheme to identify cell type-specific TdTomato+ cells in the mesenteric lymph nodes.

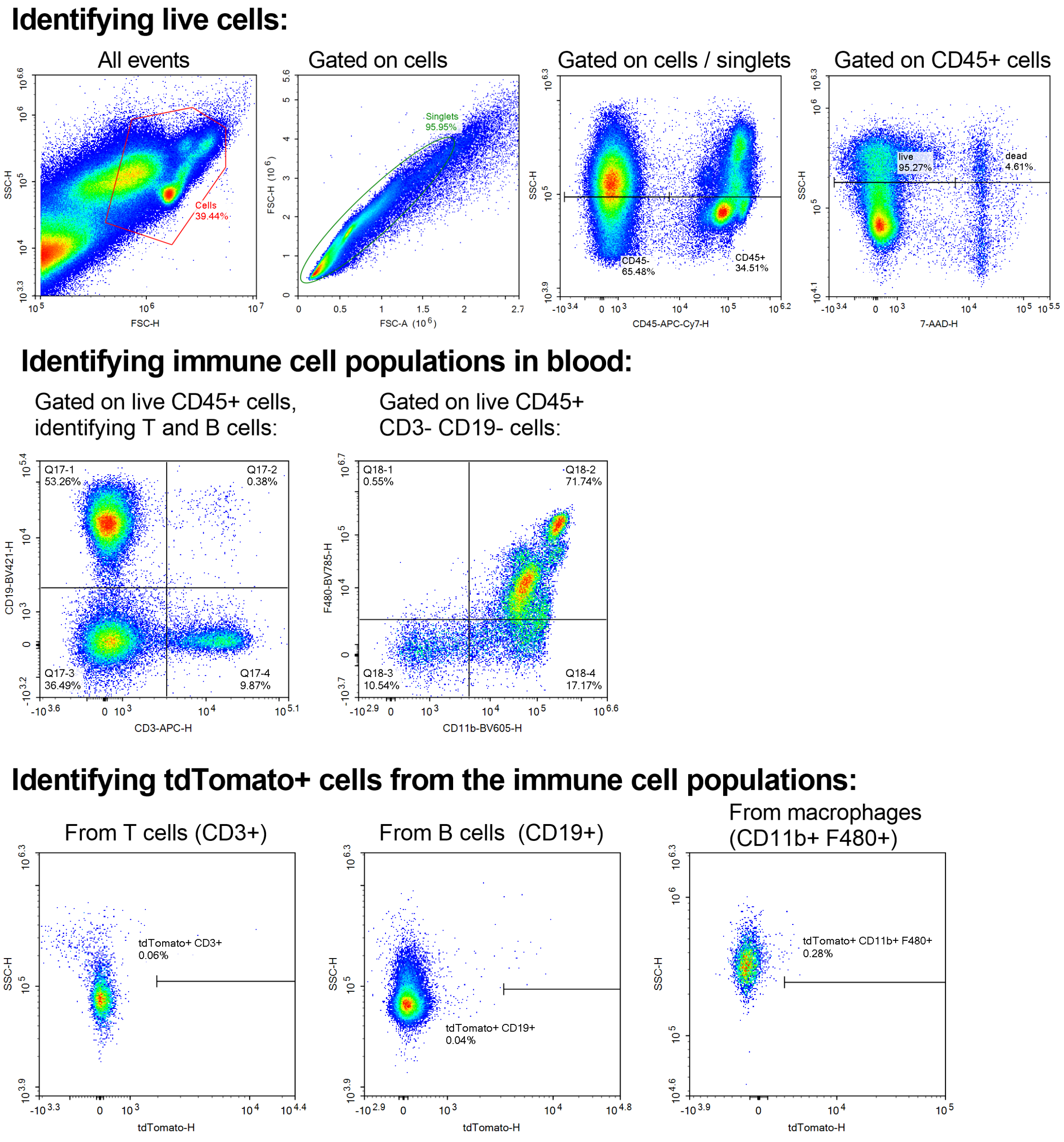

Figure S6. Gating scheme to identify cell type-specific TdTomato+ cells in blood.

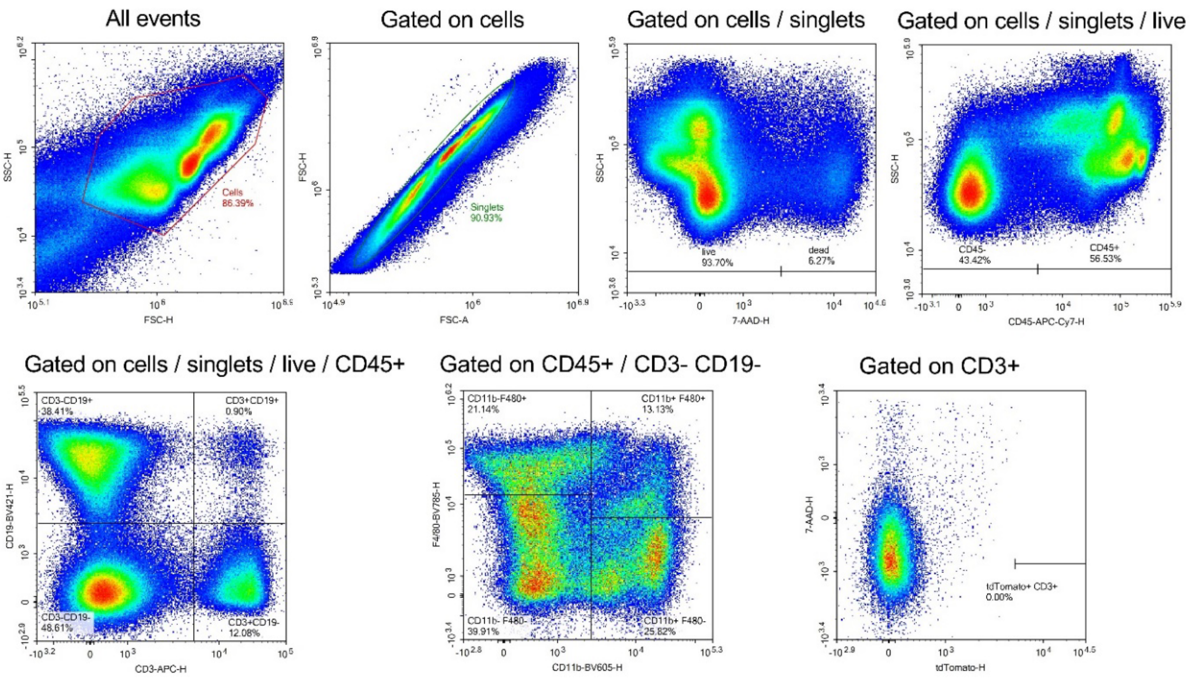

Figure S7. Gating scheme to identify cell type-specific TdTomato+ cells in the spleen.

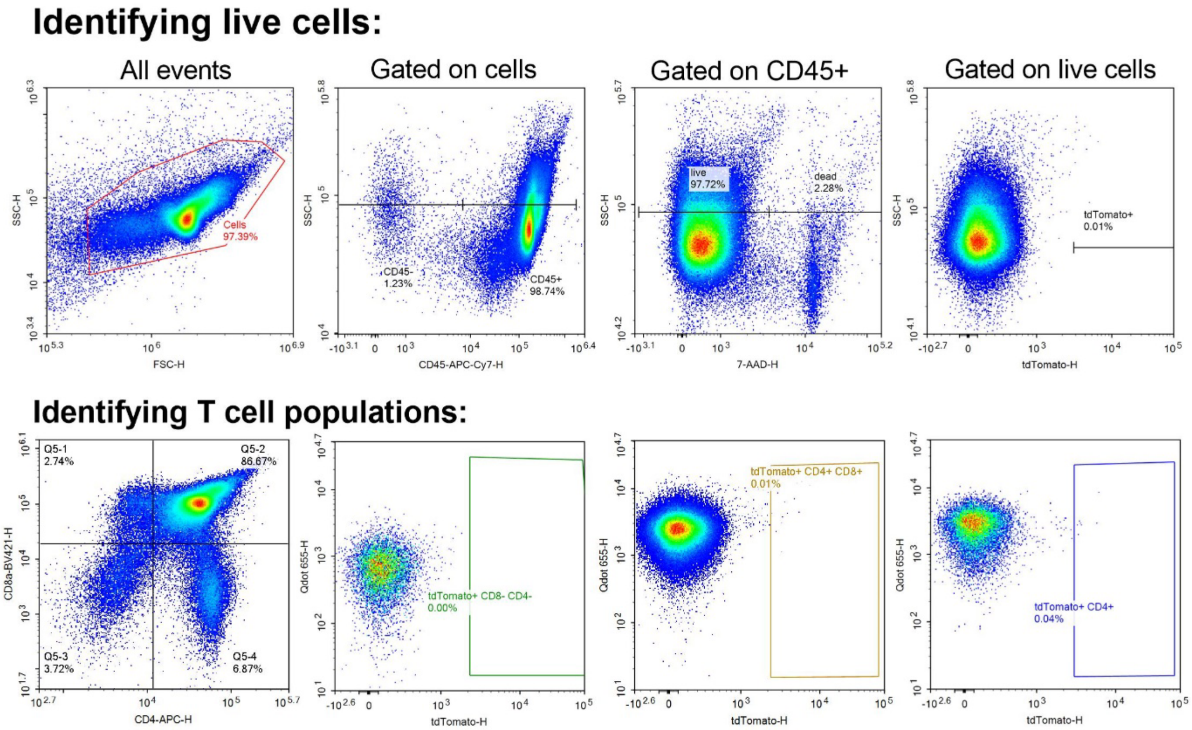

Figure S8. Gating scheme to identify cell type-specific TdTomato+ cells in the thymus.

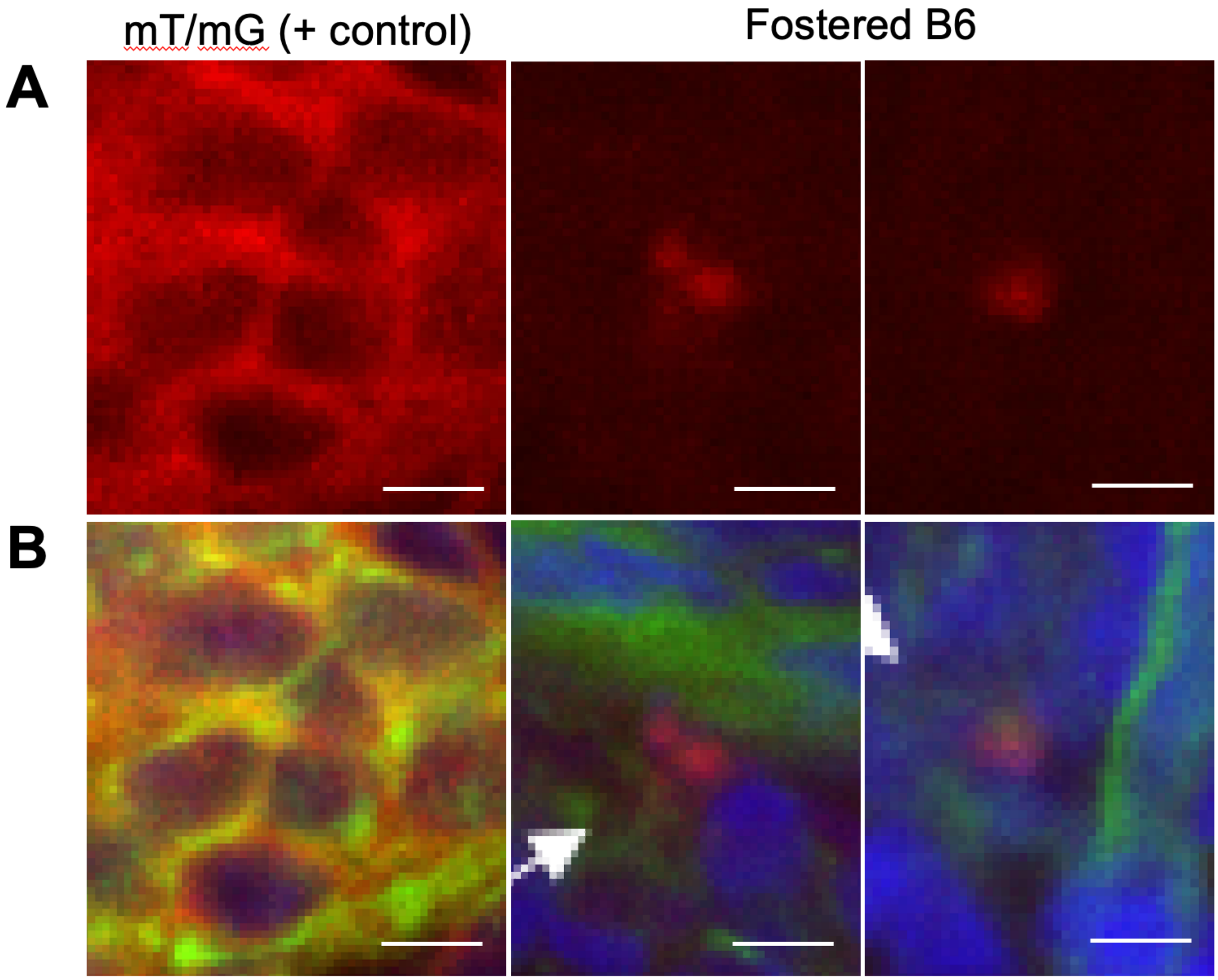

Figure S9. Milk-derived signal was visualized in pup intestinal tissue. These zoomed images from Fig. 2A show intestinal samples from positive control pups (mT/mG, left) and fostered B6 pups (middle and right). TdTomato (red) signal that is derived from milk cells is shown in **A)**, and an overlay of TdTomato, Hoescht (blue), and actin (green), is shown in **B)**. Control mice show tdTomato signal around the perimeter of the cells, whereas fostered mice have smaller areas of punctate signal. These images suggest that fostered pup intestinal samples contain milk cell fragments and not intact milk cells. Scale bars: 10 µm.

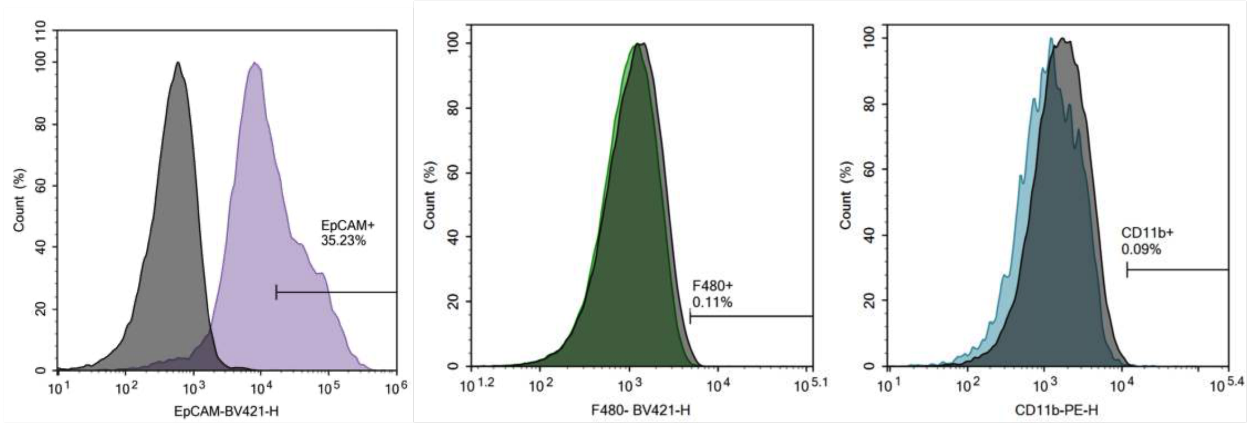

Figure S10. Milk contained epithelial cells.

Milk was freshly isolated from mT/mG dams or C57BL/6 dams (negative control) and analyzed for the presence of tdTomato+ cell types identified in foster pups by flow cytometry. Milk contained EpCAM+ (epithelial) cells but not F480+ (macrophage) or CD11b+ (monocyte) cells.

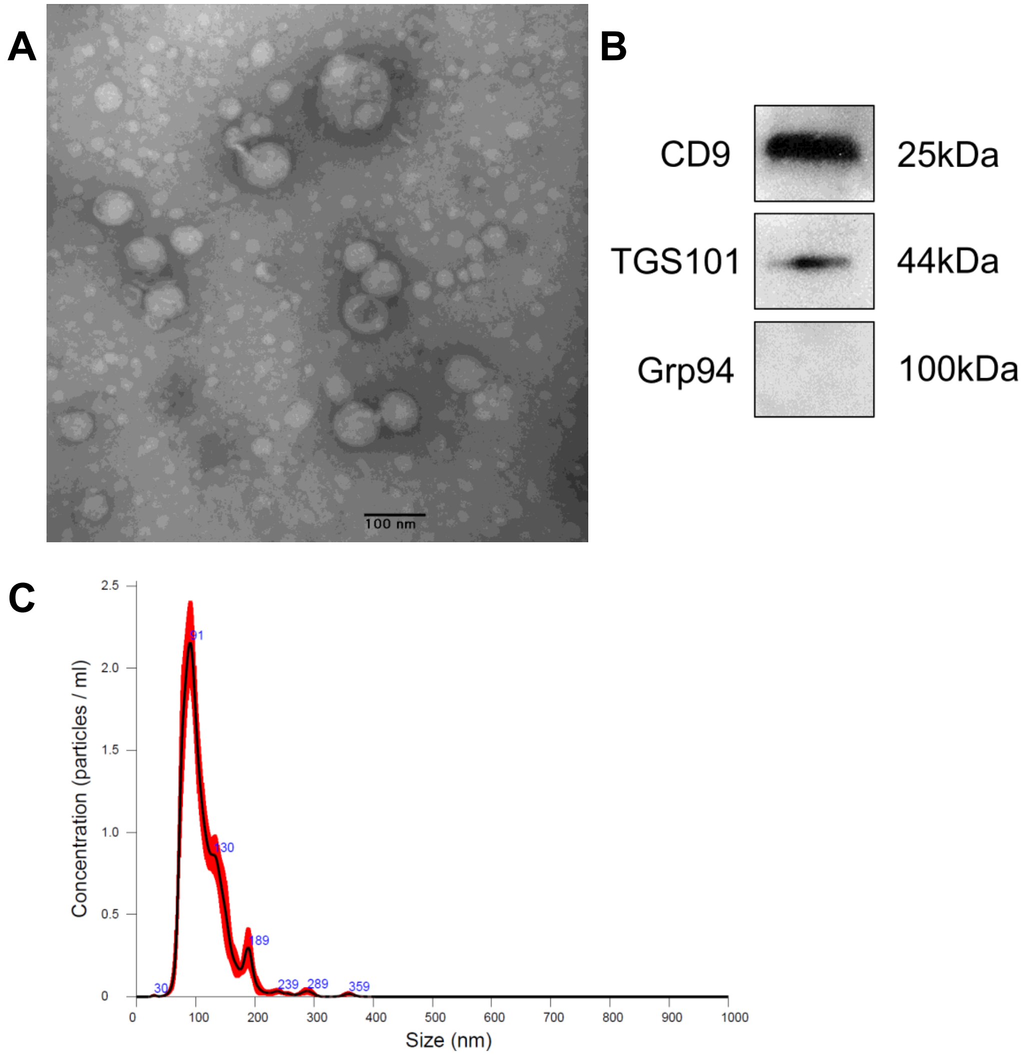

Figure S11. Characterization of extracellular vesicles from mouse milk. Milk was collected from mice by directly milking lactating dams, and extracellular vesicles were isolated by a combination of ultracentrifugation and size exclusion. **A)** TEM imaging of mouse milk EVs. Scale bar: 100 nm. **B)** Western blot of mouse milk EVs. EVs express CD9 and TGS101, commonly used markers for EVs, but do not express Grp94, a protein from the endoplasmic reticulum which should not be present in samples of extracellular vesicles. **C)** Nanoparticle tracking analysis determined the size distribution of mouse milk EVs and an average diameter of 113 nm, which is standard for EVs.
